# Acupuncture combined with repetitive transcranial magnetic stimulation for the treatment of post-stroke cognitive impairment: a systematic review and meta-analysis with trial sequential analysis

**DOI:** 10.3389/fneur.2025.1663452

**Published:** 2025-12-12

**Authors:** Xiaomeng Zhang, Jie Wang, Wen Pan, Jin Sun, Mingna He, Qinyun Wang, Peiyang Sun

**Affiliations:** 1 Second Clinical Medical College of Anhui University of Traditional Chinese Medicine, Hefei, China; 2 Second Affiliated Hospital of Anhui University of Traditional Chinese Medicine, Hefei, China

**Keywords:** stroke, repeated transcranial magnetic stimulation, acupuncture, cognitive impairment, meta-analysis, randomized controlled trial, TSA

## Abstract

**Objective:**

This study aimed to comprehensively evaluate the clinical effectiveness and safety of acupuncture combined with repetitive transcranial magnetic stimulation (rTMS) in treating post-stroke cognitive impairment (PSCI) through meta-analysis and trial sequential analysis (TSA), moreover to provide an evidence-based basis for the treatment of PSCI in clinical practice.

**Methods:**

The study conducted a comprehensive search of eight major domestic and international databases, including PubMed, Cochrane Library, Embase, Web of Science, China National Knowledge Infrastructure (CNKI), Wanfang Data, VIP and China Biology Medicine (CBM). Four English and four Chinese databases of randomized controlled trials of acupuncture combined with rTMS for the treatment of PSCI from inception until July 2025. Systematic reviews and meta-analyses were conducted based on the Cochrane systematic review method by using RevMan5.4 and Stata/MP 18.0, and trial sequential analyses were performed by TSA 0.9.

**Results:**

Sixteen RCTs involving 1,058 patients were included, including 532 patients in the experimental group and 526 patients in the control group. Meta-analysis results showed that the experimental group had a higher clinical effectiveness rate in treating patients with PSCI compared to the control group [RR = 1.29, 95% CI (1.08, 1.55), *p* = 0.005]. The experimental group significantly improved scores on several scales: Montreal Cognitive Assessment (MoCA) [MD = 2.95, 95% CI (2.37, 3.53), *p* < 0.00001], Mini-Mental State Examination (MMSE) [MD = 2.89, 95% CI (2.13, 3.64), *p* < 0.00001], LOTCA [MD = 13.61, 95% CI (6.57, 20.65), *p* = 0.0002], Modified Barthel Index (MBI) [MD = 10.86, 95% CI (7.79, 13.94), *p* < 0.00001], Activity of Daily Life (ADL) [MD = 15.33, 95% CI (10.06, 20.61), *p* < 0.00001]. Also it was found to reduced the latency of P300 in the experimental group [MD = −18.18, 95% CI (−25.76, −10.61), *p* < 0.00001] and prolonged the amplitude of P300 [MD = 1.55, 95%CI (0.71, 2.39), *p* = 0.0003]. In addition, it could increase the Brain-derived Neurotrophic Factor (BDNF) level in the blood of the patients [MD = 0.93, 95%CI (0.52, 1.35), *p* < 0.0001], and decrease the Neuron-Specific Enolase (NSE) levels [SMD = −1.26, 95% CI (−1.59, −0.93), *p* < 0.00001]. There are two studies reported the adverse events. The TSA showed that the cumulative Z value of the meta-analysis of the clinical effectiveness rate, MoCA, and MMSE scales crossed the traditional and TSA boundaries, proving reliable conclusions.

**Conclusion:**

Acupuncture combined with rTMS can improve cognitive function, regulate daily living ability, and regulate neurotransmitter levels in patients with PSCI, which is worthy recommended in the clinic. However, due to limitations in sample size, inclusion quality and incomplete reporting, it is worth noting that more rigorously designed and high-quality studies are needed to further validate these conclusions.

## 1 Introduction

Post-stroke cognitive impairment (PSCI) is a syndrome of cognitive impairment that meets the diagnostic criteria for cognitive impairment from the beginning to 6 months of an ischemic or hemorrhagic stroke, and ranges along a continuum from mild post-stroke cognitive impairment to post-stroke dementia (1, 2). Researches have shown that the probability of developing cognitive impairment 2 weeks after stroke occurrence was 24.5–34.7%, and by 3 months the probability of developing cognitive impairment reaches 24–39%, even if PSCI is prevalent in patients more than 10 years after the occurrence of the stroke (3). Most patients have an insidious onset of the disease, and even if it is not easy to detect or get attention after the onset of the disease, it will gradually progress to Post-Stroke Depression (PSD) (4), which seriously affects the survival time and quality of the patients’ life. Therefore, it is of great significance to seek for an effective treatment to implement early intervention for the patients with PSCI in clinic.

Acupuncture, as a traditional therapy, is widely used in the treatment of PSCI (5), and studies (6) have shown that acupuncture therapy can provide protection of nerves for PSCI patients by modulating the NCOA4-FTH1 pathway and regulating iron death mechanisms. Repetitive transcranial magnetic stimulation (rTMS), as a noninvasive, painless, and noninvasive electro-physiologic stimulation technique, produces painless induced currents to stimulate nerve cells in the brain. Studies (7) have also shown that rTMS can accelerate the recovery of damaged neurons, which in turn improves the cognitive function of the brain.

To date, there have been plenty of clinical randomized controlled trials of “acupuncture combined with repetitive transcranial magnetic therapy for post-stroke cognitive impairment,” but the individual reports uniformly suffer from limited sample size and insufficient evidence. The aim of this study is to conduct a meta-analysis of the published literature to systematically and rigorously evaluate the clinical efficacy and safety of this therapy, and to provide stronger evidence-based medical support for the clinical application of this therapy. In addition, traditional meta-analysis usually requires multiple tests, which can lead to increased random errors and false-positive results, but trial sequential analysis (TSA) just overcomes this deficiency (8). Meanwhile, for meta-analysis with small sample size and high heterogeneity, the application of TSA can minimize false positive results as well as judge whether statistically significant results are credible. Therefore, TSA can assess the robustness of the meta-analysis and whether more cases need to be included in further analysis to confirm the clinical results.

## 2 Methods

### 2.1 Protocol and registration

The study was duly registered with PROSPERO (registration ID CRD420251003389), serving as an international prospective registry for systematic reviews.

The conduct of this systematic review, including the primary and secondary outcomes, subgroup analyses, and statistical methods, was consistent with the pre-registered protocol. No major deviations occurred post-registration.

### 2.2 Literature search strategy

An extensive literature search was conducted using databases included four in English (Cochrane Library, PubMed, Embase, Web of Science) and four in Chinese (China Biology Medicine, VIP, Wanfang Data and CNKI). The search was spanning from the inception of the databases to July 2025. This search strategy and inclusion criteria was conducted in strict adherence to the PRISMA guidelines (9). The methodology combined subject-specific terms with free-text keywords. Search terms for Chinese databases included “post-stroke cognitive impairment,” “stroke,” “cerebral infarction,” “transcranial magnetic stimulation,” “acupuncture” and “randomized controlled.” The English search was similar. Grey literature and trial registries (e.g., ChiCTR, ClinicalTrials.gov) were excluded due to resource constraints, which may introduce language and publication bias. Future updates will expand the search to include additional languages and unpublished data. Appendix 1 detail the employed search strategies.

### 2.3 Literature inclusion criteria

(1) Literature type: randomized controlled trials of acupuncture combined with rTMS for the treatment of PSCI were included in this study, and the inclusion language was limited to Chinese and English, with no restriction on age, gender, race, or ethnicity. (2) Study subjects: standards prescribed by relevant diagnostic criteria for PSCI in the Expert Consensus on the Management of Post Stroke Cognitive Impairment 2021 (10), in which the type of stroke is not restricted and can include hemorrhagic stroke, ischemic stroke, or mixed stroke. The Mini-Mental State Examination (MMSE) and the Montreal Cognitive Assessment (MoCA) scales screened patients who rated the presence of cognitive impairment. Commonly applied thresholds for cognitive impairment in the included studies were MMSE ≤26 or MoCA ≤25, adjusted for education level where applicable. (3) Age ≥18 years, and no upper age limit; vital signs were basically normal, and the patients were able to cooperate with the treatment and scale assessments. (4) Interventions: The trial was divided into a experimental group and a control group. In addition to the conventional basic treatment (include neuroprotective agents, antiplatelet therapy, management of vascular risk factors, or standard rehabilitation training, etc.), the experimental group received additional acupuncture (no limit to the method of acupuncture) combined with rTMS (low-frequency or high-frequency), while the control group received one or more of the following: conventional treatment alone or conventional treatment combine with rTMS. Studies combining acupuncture or rTMS with other traditional Chinese medicine therapies (e.g., herbal medicine, tuina) were excluded. (5) Study type: randomized controlled trial. (6) The results of the study data must be complete and valid. (7) Outcome measures: primary outcome measures included the MMSE scale and MoCA scales; secondary outcome measures included the total clinical effectiveness, other life ability scales and serologic indicators.

### 2.4 Literature exclusion criteria

(1) The experimental or control group included other traditional Chinese medicine (TCM) treatments, such as herbal therapy or massage therapy, etc. (2) The study subjects included in the literature did not meet the diagnostic criteria for PSCI or did not have a clear diagnostic criterion. (3) Cognitive impairment caused by other diseases. (4) The outcome measures were not available. (5) Duplicated published literature. (6) Animal-type experiments, reviews, dissertations, conference papers, famous experts’ experience, no full-text literature or no baseline information literature, etc. (7) Quasi-randomized trials (e.g., those using allocation by birth date, hospital record number) and studies that did not describe an appropriate random sequence generation method.

### 2.5 Literature screening and data extraction

Literature screening was performed by two researchers according to the inclusion and exclusion criteria. Discrepancies were resolved through discussion between the two reviewers or, if necessary, consultation with a third party. Literature information extraction was performed using a standardized Excel template, and the extracted information included: name of the first author, year of publication, sample size of the two groups, duration of treatment, age, method of random allocation, interventions, and outcome measures. Additionally, the reference lists of all included studies and relevant systematic reviews were manually screened to identify any potentially eligible studies that may have been missed in the database search.

### 2.6 Quality evaluation

All included studies were randomized controlled trials, and risk of bias was assessed for included studies using the Cochrane RoB2.0 Risk of Bias Tool (11). The tool assesses potential bias in six key areas, including random sequence generation, allocation concealment, blinding of participants and personnel, blinding of outcome assessment, completeness of outcome data, selective reporting, and other potential biases. The risk was low if the methodology was appropriate and well-founded, otherwise it was high, and the risk was unknown if relevant information was not mentioned. To ensure rigorous methodological standards, the risk of bias for each included study was assessed independently by two independent researchers, with a third-party researcher resolving any discrepancies or disagreements identified during the independent assessment to ensure impartiality of the assessment. Two trained reviewers (JS and WP) independently assessed the included studies using RoB 2 and calculated the intra-class correlation coefficient (ICC) to conduct a consistency test. If the consistency reached at least 80%, a formal assessment was conducted. Any discrepancies were resolved through discussion with a third reviewer (XMZ).

The GRADE tool was utilized to evaluate the overall quality of the evidence, which is categorized into four levels: high (A), medium (B), low (C), and extremely low (D). For all outcome measures, the evidence quality ranged from moderate to very low. During the evaluation process, the decision to downgrade the evidence was based on five key factors: research limitations, inconsistency, indirectness, imprecision, and publication bias. GRADE criteria were applied independently by two reviewers, with disagreements resolved by a third. Downgrading reasons included risk of bias (unblinded designs), inconsistency (*I*^2^ > 50%), and imprecision (small sample size).

### 2.7 Statistical methods

Meta-analyses were performed using Review Manager (RevMan) 5.4, with odds ratios (OR) or risk ratios (RR) as effect indicators for dichotomous data, and mean difference (MD) or standardized mean difference (SMD) as effect indicators for continuous data. Confidence intervals (CIs) were calculated at the 95% level, and heterogeneity was assessed using the *I*^2^ statistic; when *p* > 0.10 and *I*^2^ ≤ 50%, it indicated less heterogeneity, and a fixed-effects model was used; whereas, when *p* ≤ 0.10 and *I*^2^ > 50%, it indicated more heterogeneity, and a random-effects model was used, and subgroups or sensitivity analyses were performed as needed. The results of the meta-analyses were presented as forest plots. When the analysis included more than eight outcome indicators, the presence of publication bias was assessed qualitatively by funnel plots and quantitatively by applying Stata/MP 18.0 software using Begge’s and Egger’s tests, and when publication bias existed, further quantitative analyses were performed by the clipping method to assess the robustness of the results of meta-analysis. TSA0.9.5.10 beta was used to perform trial sequential analysis (TSA) on clinical effectiveness rate, MoCA scale, and MMSE scale to reduce the occurrence of random errors and to determine the reliability of the conclusions. The sample size was used as the expected information value, and the analysis was performed with a significance level of *α* = 0.05 for type I error, power of *β* = 0.2 for type II error, and a statistical efficacy of 80% to minimize random errors and ascertain the reliability of the results. Additionally, an estimation of the required sample size (RIS) for meta-analysis was also carried out.

For trials with multiple intervention arms, only the relevant arms (acupuncture combined with rTMS vs. control) were included to avoid double-counting of participants. If a trial reported multiple time points for the same outcome, only data from the final follow-up assessment were included in the primary analysis.

## 3 Results

### 3.1 Literature screening

**Figure 1:**
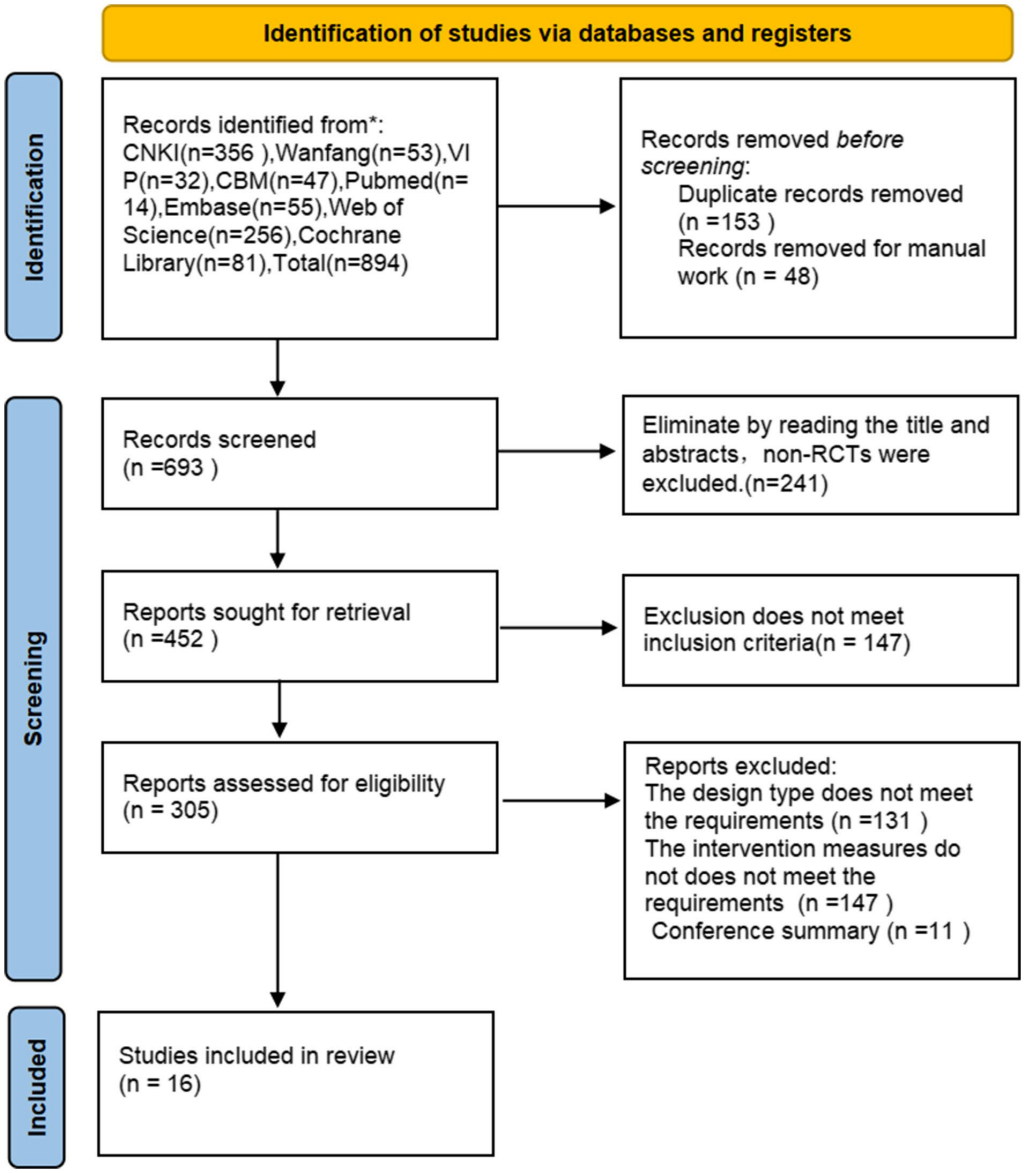
Literature screening process for acupuncture combined with rTMS in the treatment of PSCI.

According to the search strategy, 820 literatures were initially retrieved. Finally 16 literatures, including 1,058 patients, were included through removing duplicates using Endnote software, reading the title, abstract, and full text, and excluding non-compliant documents, and the specific screening process of the documents. The literature screening process is illustrated in Figure 1.

### 3.2 Basic information on included studies

The sixteen studies were RCTs involving 1,058 patients, including 532 in the experimental group and 526 in the control group. All studies were conducted in China and published between 2015 and 2024, with sample sizes ranging from 30 to 106 cases and duration of treatment ranging from 2 to 8 weeks. There are six studies included patients with ischemic stroke, and ten (12–21) studies of stroke type were unrestricted, including ischemic and hemorrhagic strokes. The interventions in the experimental group included electro-acupuncture, scalp acupuncture, specific acupuncture and conventional acupuncture. There are four (15, 18, 21, 22) studies in the control group used only conventional therapies, including conventional drug therapies such as nutritive neurologic and microcirculation improvement, as well as rehabilitation interventions. There were no statistically significant differences in gender, mean age, and duration of disease between groups for all included patients (*p* > 0.05), and they were comparable. In the 16 study outcome indicators, exist five (12, 13, 15, 23, 24) clinical effectiveness rate comparisons, thirteen (12, 15–22, 24–27) MoCA scale comparisons, nine (13–17, 20, 22, 24, 26) MMSE scale comparisons, three (13, 16, 20) LOTCA scale comparisons, three (16, 18, 19) P300 potential latency and wave amplitude comparisons, two (24, 25) NSE comparisons, three BNDF (24, 25, 27) comparisons, four (12, 14, 16, 19) MBI comparisons, two (15, 21) ADL comparisons, and two (12, 26) study reported adverse effects. The basic characteristics of the studies were comparable, detailed in Table 1.

**Table 1:** Basic information of the included literature.

Study	Stroke type	Sample size	Gender (M/F)	Average age	Course	Intervention	Outcomes
Lei X. L. (2021) (12)	Hemorrhagic stroke and ischemic stroke	31/30	E:11:20 C:17:13	E:58.03 ± 5.53 C:57.10 ± 5.80	8 weeks	E:EA+C C:R+rTMS	MoCA, MBI, Clinical effectiveness rate
Huang H. Q. (2022) (23)	Ischemic stroke	50/49	E:19:31 C:19:30	E:59.07 ± 11.05 C:58.44 ± 10.42	4 weeks	E:EA+C C:rTMS	Clinical effectiveness rate
Han J. (2024) (25)	Ischemic stroke	53/53	E:30:23 C:29:24	E:65.33 ± 3.51 C:65.17 ± 3.61	4 weeks	E:EA+C C:R+rTMS	MoCA, BDNF, NSE
Gao L. J. (2019) (13)	Hemorrhagic stroke and ischemic stroke	30/30	E:17:13 C:16:14	E:64.33 ± 9.07 C:61.80 ± 13.39	4 weeks	E:CA+C C:R+rTMS	LOTCA, MMSE, Clinical effectiveness rate
Liu L. (2019) (26)	Ischemic stroke	41/39	E:21:20 C:20:19	E:55.00 ± 13.84 C:54.31 ± 13.31	4 weeks	E:SA+C C:rTMS	MoCA, MMSE
Han C. Y. (2018) (14)	Hemorrhagic stroke and ischemic stroke	15/15	E:11:4 C:11:4	E:60.3 ± 8.1 C:61.5 ± 8.0	8 weeks	E:SA+C C:rTMS	MMSE, MBI
Deng Z. X.(2015) (15)	Hemorrhagic stroke and ischemic stroke	21/21	25:17	62.24 ± 10.25	4 weeks	E:SA+rTMS+C C:R	MoCA, MMSE, ADL, Clinical effectiveness rate
Shang Y. X. (2022) (27)	Ischemic stroke	30/30	E:18:12 C:19:11	E:57 ± 6.8 C:56 ± 6.6	4 weeks	E:SA + C C:rTMS	MoCA, BDNF
Su J. J.(2024) (16)	Hemorrhagic stroke and ischemic stroke	30/30	E13:17 C:14:16	E:64.10 ± 13.08 C:64.17 ± 10.42	2 weeks	E:Xingnao Kaiqiao+C C:rTMS	MMSE, MoCA, LOTCA, MBI, P300
Lin Y. Y. (2019) (24)	Ischemic stroke	33/33	E:19:14 C:18:15	E:63.53 ± 9.01 C:65.15 ± 9.26	2 weeks	E:CA+C C:rTMS	MoCA, MMSE, BDNF, NSE, Clinical effectiveness rate
Sun H. H. (2019) (18)	Hemorrhagic stroke and ischemic stroke	50/50	E:31:19 C:28:22	E:57.79 ± 7.04 C:56.14 ± 8.37	8 weeks	E:CA+rTMS+C C:R	MoCA, P300
Yu T. Y.(2018) (21)	Hemorrhagic stroke and ischemic stroke	30/30	E:18:12 C:16:14	E:63. 77 ± 2. 05 C:64. 04 ± 1. 83	4 weeks	E:CA+rTMS+C C:R	MoCA, ADL
Zhao N. N.(2023) (19)	Hemorrhagic stroke and ischemic stroke	41/39	E:21:20 C:20:19	E:62 ± 7 C:63 ± 8	4 weeks	E:Xingnao Kaiqiao+C C:rTMS	MoCA, MBI, P300
Dan Z. J. (2016) (17)	Hemorrhagic stroke and ischemic stroke	27/27	E:20:7 C:19:8	E:51.28 ± 12.78 C:52.07 ± 10.66	4 weeks	E:Tongdu Xingshen+C C:rTMS	MMSE, MoCA
Li Z. B.(2021) (22)	Ischemic stroke	30/30	E:17:13 C:18:12	E:57.6 ± 5.6 C:59.1 ± 7.1	4 weeks	E:EA+rTMS+C C:R	MMSE, MoCA
Wang J. J. (2021) (20)	Hemorrhagic stroke and ischemic stroke	20/20	E:9:11 C:8:12	E:53.78 ± 6.28 C:54.32 ± 5.46	8 weeks	E:CA+C C:R+rTMS	MoCA, MMSE, LOTCA

E, experimental group; C, control group; CA, conventional acupuncture; SA, scalp acupuncture; R, routine therapy; EA, electro-acupuncture. MMSE, Mini-Mental State Examination; MoCA, Montreal Cognitive Assessment; ADL, Activity of Daily Life. NSE, Neuron-Specific Enolase; BDNF, Brain-derived Neurotrophic Factor MBI, Modified Barthel Index. LOTCA: Loewenstein Occupational Therapy Cognitive Assessment.

### 3.3 Quality assessment of included studies

The ICC value for the RoB 2 assessment by the two reviewers was 0.862, indicating excellent consistency.

**Figure 2:**
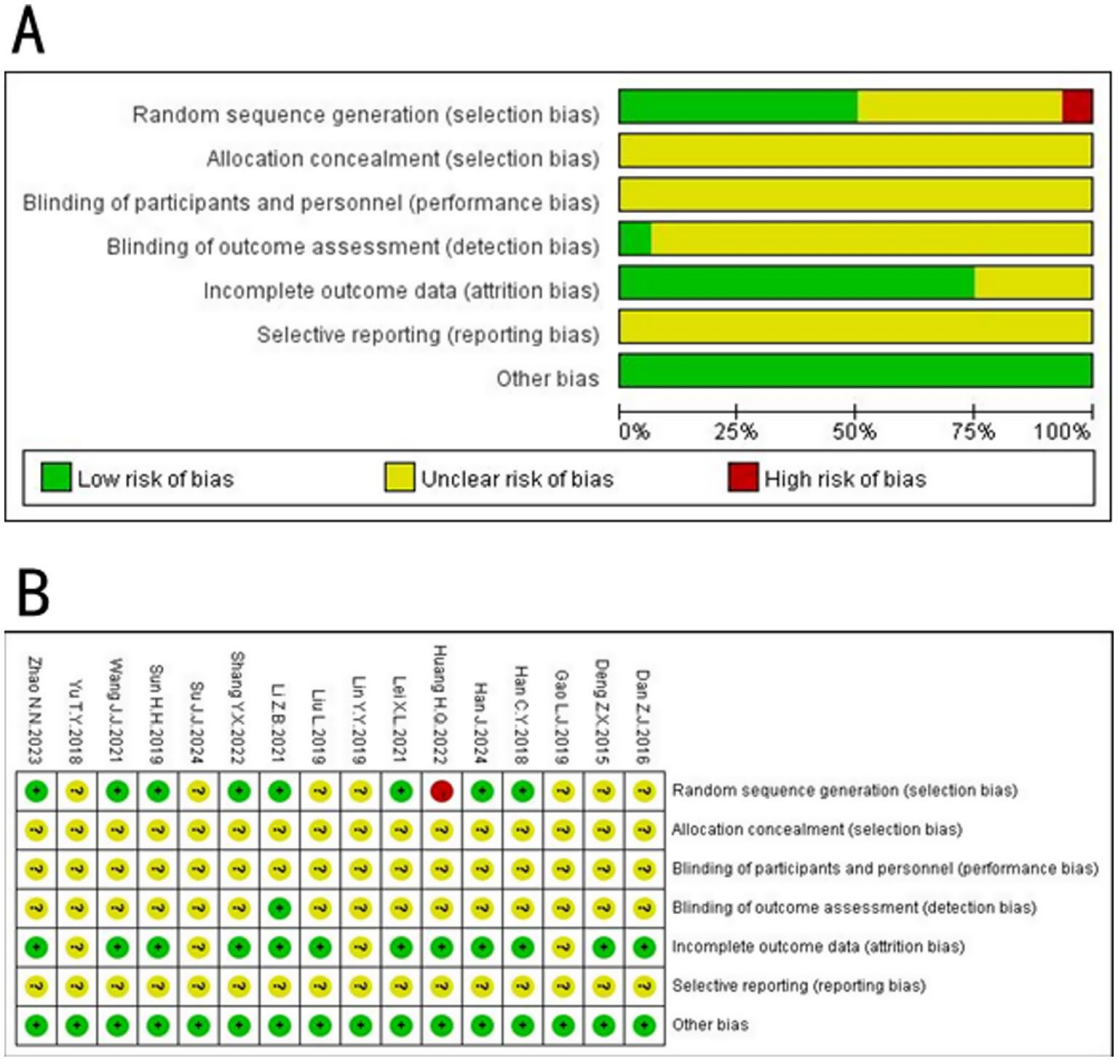
Risk of bias graph **(A)** and summary **(B)** were summarized for all included studies.

In the 16 included studies, eight studies (12, 14, 18–20, 22, 25, 27) employed the random number table method for randomization and were rated as “low risk,” seven studies (13, 15–17, 21, 24, 26) mentioned randomization only and did not specifically account for grouping methods and were rated as “unknown risk,” and one (23) did not mention grouping methods and was rated as “unknown risk.” None of the 16 studies reported allocation concealment, which was assessed as “unknown risk”; none of the included literature was blinded to participants, which was assessed as “unknown risk”; only one (22) study reported blinding of outcome indicator raters, which was assessed as “low risk,” and the rest of the studies rated the risk as “unknown.” Four (13, 16, 21, 24) studies did not specify the number of cases when reporting the outcome indicator and assessed it as “unknown risk.” For selective reporting, the risk was assessed as “unknown” because raw data were not available. All data were statistically comparable at baseline and no other potential sources of bias were described, so they were assessed as “low risk.” The risk of bias graph and the risk of bias summary graph are shown in Figure 2. Details of the GRADE scores for the included studies are provided in Table 2.

**Table 2:** GRADE scores for the included studies.

Outcomes	Number of participants (studies)	Effect size (95% CI) quality assessment	Quality assessment					Level of evidence
			Risk of bias	Inconsistency	Indirectness	Imprecision	Other considerations (publication bias)	
Clinical effectiveness rate	349 (five studies)	RR = 0.77, 95% CI (0.65, 0.93)	Serious (1)	Serious (2)	Not serious	Not serious	Not found	C
MoCA scores	869 (thirteen studies)	MD = 2.95, 95% CI (2.37, 3.53)	Serious (1)	Serious (2)	Not serious	Not serious	Not found	C
MMSE scores	492 (nine studies)	MD = 2.89, 95% CI (2.13, 3.64)	Serious (1)	Serious (2)	Not serious	Not serious	Not found	C
LOTCA	120 (three studies)	MD = 13.61, 95% CI (6.57, 20.65)	Serious (1)	Serious (2)	Not serious	Serious (3)	Not found	D
The latency of P300	240 (three studies)	MD = −18.18, 95% CI (−25.76, −10.61)	Serious (1)	Serious (2)	Not serious	Serious (3)	Not found	D
The amplitude of P300	240 (three studies)	MD = 1.55, 95% CI (0.71, 2.39)	Serious (1)	Serious (2)	Not serious	Serious (3)	Not found	D
NSE	172 (two studies)	SMD = −1.26, 95% CI (−1.59, −0.93)	Serious (1)	Not serious	Not serious	Serious (3)	Not found	C
BDNF	232 (three studies)	SMD = 0.93. 95% CI (0.52, 1.35)	Serious (1)	Serious (2)	Not serious	Serious (3)	Not found	D
MBI	231 (four studies)	MD = 10.86, 95% CI (7.79, 13.94)	Serious (1)	Not serious	Not serious	Serious (3)	Not found	C
ADL	101 (two studies)	MD = 15.33, 95% CI (10.06, 20.61)	Serious (1)	Not serious	Not serious	Serious (3)	Not found	C

Reduced by one level: (1) Some studies have unclear reports on randomized methods, lack of blinding participants or personnel and allocation concealment. (2) Heterogeneity among included studies (*p* < 0.05 or *I*^2^ ≥ 50%). (3) The relatively small sample size (≤4 samples or <300 participants) leads to a wider confidence interval, which affects accuracy.

### 3.4 Meta-analysis results

#### 3.4.1 Clinical effectiveness rate

**Figure 3:**
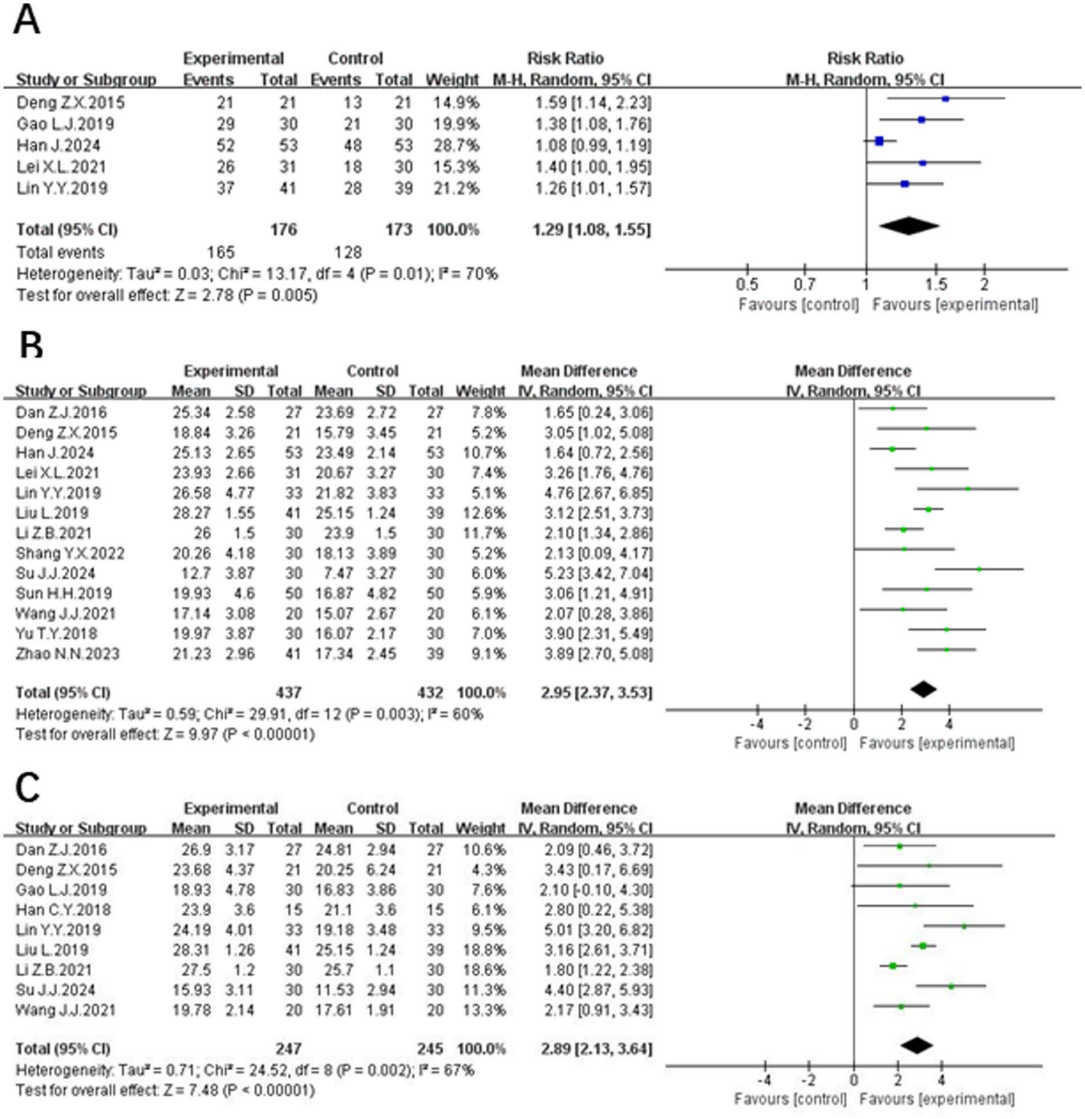
The forest plot of the effectiveness **(A)**, MoCA scores **(B)** and MMSE scores **(C)** of acupuncture combined with rTMS in the treatment of PSCI.

Five studies (12, 13, 15, 23, 24), involving 349 patients, reported the clinical efficacy. The studies exhibited substantial heterogeneity (*p* = 0.01, *I*^2^ = 70%), which was analyzed using random effects modeling. The results of meta-analysis showed that the clinical effectiveness rate was higher in the experimental group compared with the control group, and the difference was statistically significant [RR = 1.29, 95% CI (1.08, 1.55), *p* = 0.005], as illustrated in Figure 3.

#### 3.4.2 MoCA scores

Thirteen studies (12, 15–22, 24–27), involving 869 patients, reported on MoCA scores. Twelve of the studies used a total score system, while Sun (18) counted the individual separate scores in detail. These studies showed significant heterogeneity (*p* = 0.003, *I*^2^ = 60%), necessitating a random-effects meta-analysis. The results of meta-analysis indicated that the MoCA scale scores of the experimental group were significantly higher than those of the control group, and the difference was statistically significant [MD = 2.95, 95% CI (2.37, 3.53), *p* < 0.00001], as shown in Figure 3.

#### 3.4.3 MMSE scores

Nine studies (13–17, 20, 22, 24, 26), involving 492 patients, reported on MMSE scores. These studies demonstrated heterogeneity (*p* = 0.002, *I*^2^ = 67%) and was analyzed using a random effects model. The results of meta-analysis b showed that the MMSE scale scores of the experimental group were higher than those of the control group, and the difference was statistically significant [MD = 2.89, 95% CI (2.13, 3.64), *p* < 0.00001], illustrated in Figure 3.

#### 3.4.4 Loewenstein occupational therapy cognitive assessment (LOTCA)

**Figure 4:**
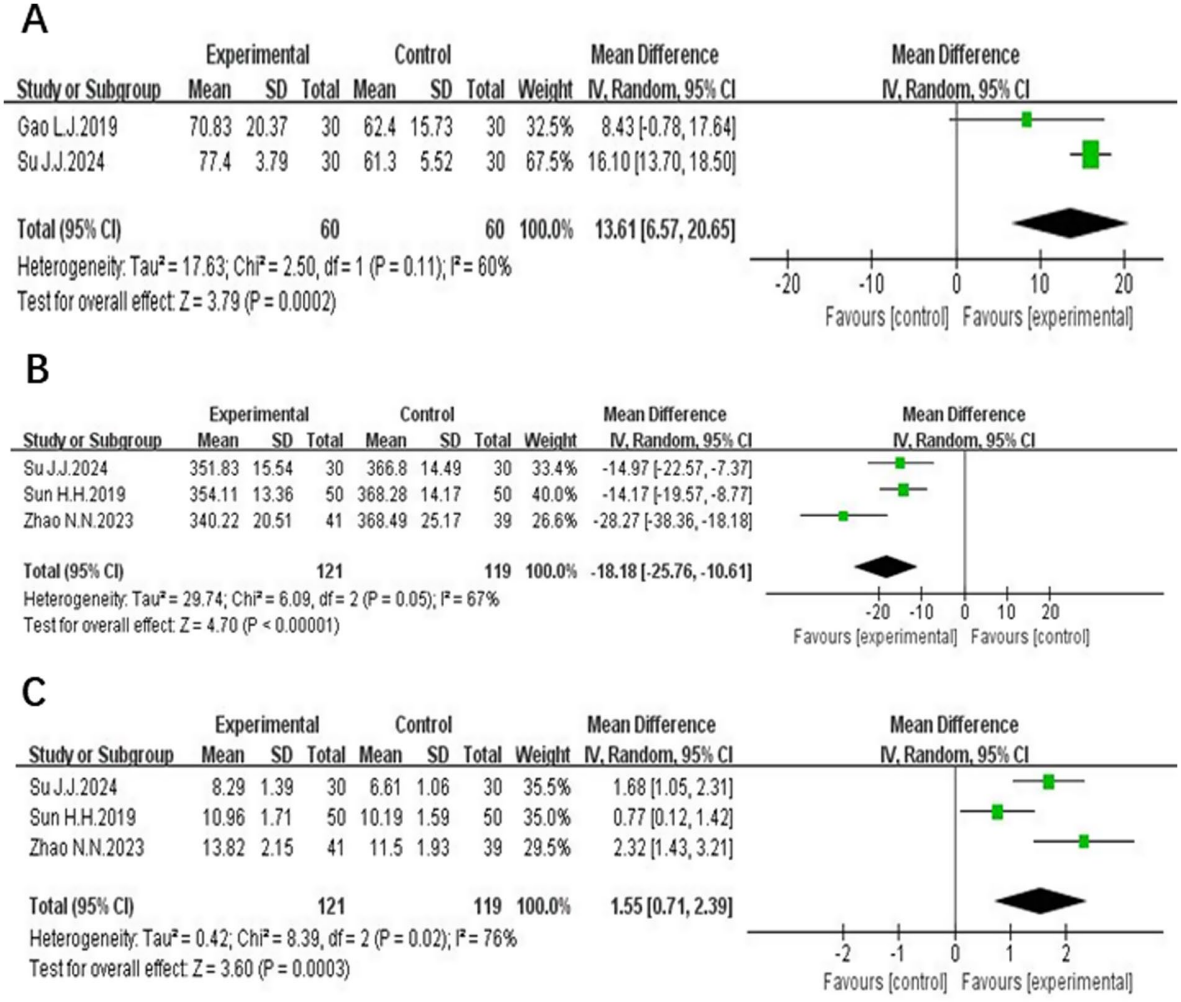
The forest plot of the total LOTCA scores **(A)**, the latency of P300 **(B)** and the amplitude of P300 **(C)** of acupuncture combined with rTMS in the treatment of PSCI.

Three studies (13, 16, 20), involving 120 patients, reported on LOTCA scores. Two of them (13, 16) used the total LOTCA scores and exhibited heterogeneity (*p* = 0.11, *I*^2^ = 60%). A random-effects meta-analysis indicated that the LOTCA scores in the experimental group were significantly higher than those in the control group, with a statistically significant difference [MD = 13.61, 95% CI (6.57, 20.65), *p* = 0.0002]. The scores of Wang Jingjing (20) used each item separately and found that orientation, optomotor organization, visual perception, thought manipulation, spatial perception and movement use were significantly higher in the treatment group than in the pre-treatment and control groups (*p* < 0.05), shown in Figure 4.

#### 3.4.5 The latency and the amplitude of P300

Three studies (16, 18, 19) discussed the latency and the amplitude of P300, enrolling 240 subjects, 121 in the experimental group and 119 in the control group. The heterogeneity between the latency of P300 studies was large (*p* = 0.05, *I*^2^ = 67%) and was analyzed using a random effects model. The results indicated that acupuncture combined with rTMS therapy significantly reduced the latency of P300 in PSCI patients compared with the control group, and the difference was statistically significant [MD = −18.18, 95% CI (−25.76, −10.61), *P <* 0.00001]. The results are depicted in Figure 4. There was heterogeneity between studies of the amplitude of P300 (*p* = 0.02, *I*^2^ = 76%)and the results of meta-analysis by random effects model showed, indicating that patients in the treatment group had a prolongation of the amplitude of P300 compared with the control group, and the difference was statistically significant [MD = 1.55, 95% CI (0.71, 2.39), *p* = 0.0003]. The results are depicted in Figure 4.

#### 3.4.6 Neuron specific enolase (NSE)

**Figure 5:**
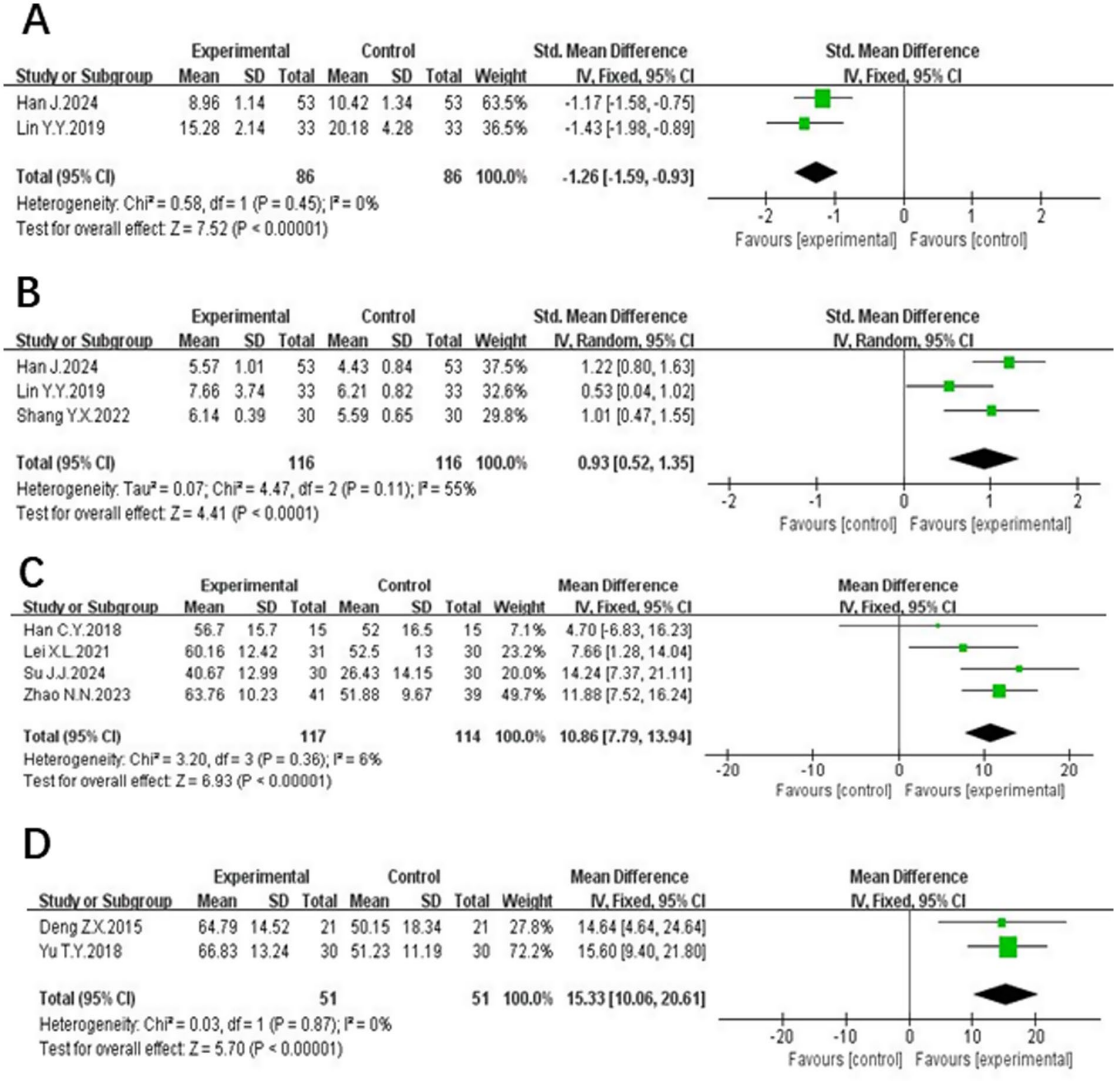
The forest plot of NSE **(A)**, BDNF **(B)**, MBI **(C)** and ADL **(D)** of acupuncture combined with rTMS in the treatment of PSCI.

Two studies (24, 25) evaluated NSE, involving a total of 172 subjects. The NSE units of the two studies were not the same, so the standardized mean difference (SMD)was used for meta-analysis. These studies demonstrated homogeneity (*p* = 0.45, *I*^2^ = 0%) and a fixed-effect meta-analysis showed that the NSE level of the test group was lower than that of the control group and the difference was statistically significant [SMD = −1.26, 95% CI (−1.59, −0.93), *p* < 0.00001], as detailed in Figure 5.

#### 3.4.7 Brain-derived neurotrophic factor (BDNF)

Three studies (24, 25, 27) examined BDNF, involving a total of 232 participants. The BDNF units of the three studies were not the same, so the standardized mean difference SMD was used for the meta-analysis, and there was a heterogeneity between the studies (*p* = 0.11, *I*^2^ = 55%). The results of the meta-analysis by the random-effects model showed that the BDNF level of the experimental group was higher than that of the control group, and the difference was statistically significant [SMD = 0.93. 95% CI (0.52, 1.35), *p* < 0.0001], shown in Figure 5.

#### 3.4.8 Modified Barthel Index (MBI)

Four studies (12, 14, 16, 19) comprising 231 patients, reported MBI. Homogeneity was noted (*p* = 0.36, *I*^2^ = 6%), and a fixed-effect meta-analysis revealed that the modified barthel index scores of the experimental group were higher than those of the control group, and the difference was statistically significant [MD = 10.86, 95% CI (7.79, 13.94), *p* < 0.00001]. These findings are presented in Figure 5.

#### 3.4.9 Activity of daily life (ADL)

Two studies (15, 21) involving 101 patients reported on ADL scores, with 51 cases in the experimental group and 51 cases in the control group. These studies demonstrated homogeneity (*p* = 0.87, *I*^2^ = 0%), and a fixed-effect meta-analysis showed that ADL scores in the experimental group was higher than that of the control group, and the difference was statistically significant [MD = 15.33, 95% CI (10.06, 20.61), *p* < 0.00001], illustrated in Figure 5.

#### 3.4.10 Adverse event reporting

Two studies mentioned adverse effects, one (26) showed that no adverse effects occurred in both the experimental and control groups. One (12) reported one case of panic in the experimental group using electro-acupuncture combined with rTMS, which was tolerable and did not show any significant abnormality on relevant examination. Two studies of mild dizziness in the control group using rTMS.

### 3.5 Subgroup analysis of MoCA scale

#### 3.5.1 Subgroup analysis by treatment course

**Figure 6:**
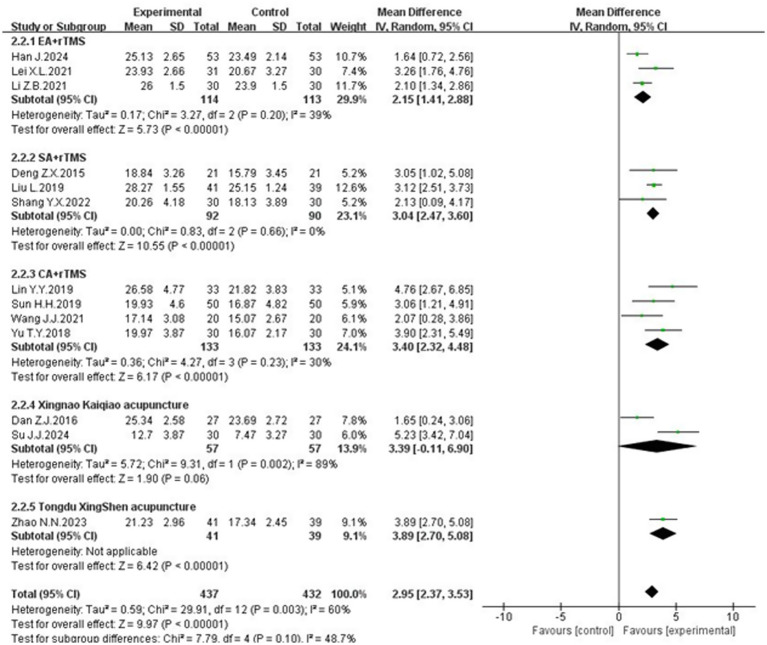
The forest plot of the MoCA scores based on the difference in course.

Given that the heterogeneity in MoCA scale scores across the thirteen studies might be attributed to variations in treatment course, a subgroup analysis was conducted, categorizing the treatment duration into 2, 4, and 8 weeks. The results indicated a significant reduction in heterogeneity for the 2-week and 8-week treatment groups. Among patients with a treatment course of 2 weeks, the results of the heterogeneity test showed that *p* = 0.74, *I*^2^ = 0%, and the combined effect size showed that [MD = 5.03, 95% CI (3.66, 6.40), *p* < 0.00001]; among patients with a treatment period of 4 weeks, the results of the heterogeneity test showed that *p* = 0.01, *I*^2^ = 61%, and the combined effect size showed that [MD = 2.65, 95%CI (2.00, 3.29), *P <* 0.00001]; among patients with a treatment period of 8 weeks, the test for heterogeneity showed: *p* = 0.59, *I*^2^ = 0%, and the combined effect size showed: [MD = 2.85, 95% CI (1.87, 3.82), *p* < 0.00001]. The MoCA scale scores were significantly higher in the experimental group compared to the control group across all treatment duration subgroups, with statistically significant differences (Figure 6). The 4-week treatment course emerged as a potential critical time point for acupuncture combined with rTMS in treating PSCI, which may explain the observed heterogeneity. Additionally, the heterogeneity could also be influenced by variations in acupuncture techniques and control group interventions.

#### 3.5.2 Subgroup analysis by intervention methods

**Figure 7:**
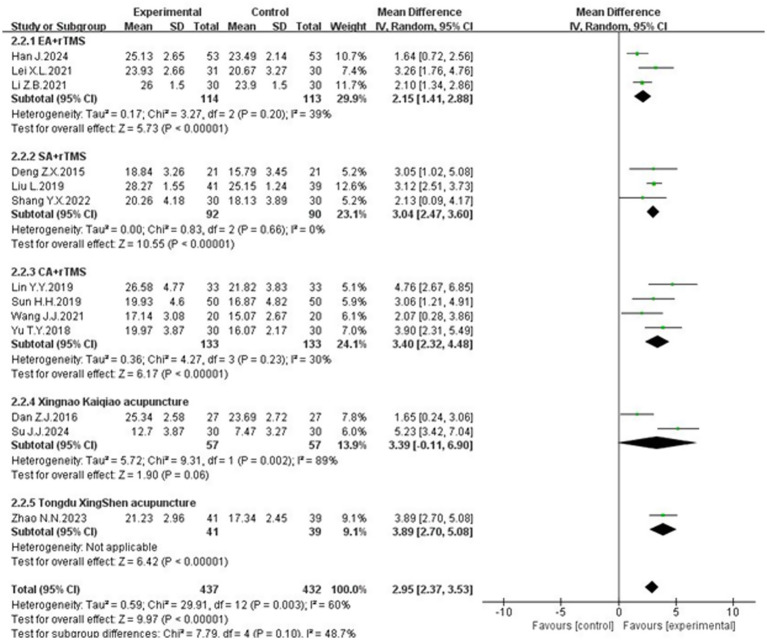
The forest plot of the MoCA scores based on the difference in intervention methods.

The heterogeneity in MoCA scale scores might also stem from differences in acupuncture modalities. Therefore, interventions were categorized into following groups:electro-acupuncture + rTMS, scalp acupuncture + rTMS, conventional acupuncture + rTMS and specific acupuncture + rTMS. The analysis revealed a significant reduction in heterogeneity for the electro-acupuncture, scalp acupuncture and conventional acupuncture groups. Electro-acupuncture + rTMS group: Heterogeneity test results: *p* = 0.20, *I*^2^ = 39%. The combined effect size was [MD = 2.15, 95% CI (1.41, 2.88), *p* < 0.00001]. Scalp acupuncture + rTMS group: Heterogeneity test results: *p* = 0.66, *I*^2^ = 0%. The combined effect size was [MD = 3.04, 95% CI (2.47, 3.60), *p* < 0.00001]. Conventional acupuncture + rTMS group: Heterogeneity test results: *p* = 0.23, *I*^2^ = 30%. The combined effect size was [MD = 3.40, 95% CI (2.32, 4.48), *p* < 0.00001]. Specific acupuncture group include Xingnao Kaiqiao acupuncture and Tongdu Xingshen acupuncture. The results show that *p* < 0.00001 in the Tongdu Xingshen acupuncture group. All the above groups demonstrated significantly higher MoCA scale scores compared to the control group. However, the Xingnao Kaiqiao acupuncture exhibited high heterogeneity (*p* = 0.06, *I*^2^ = 89%), with a combined effect size of [MD = 3.39, 95% CI (−0.11, 6.90), *p* = 0.06], indicating no statistically significant difference (Figure 7). The variability in outcomes for Xingnao Kaiqiao acupuncture may be due to the high level of skill required for its techniques, particularly the emphasis on “De qi” leading to inconsistent standardization among practitioners.

### 3.6 Publication bias assessment

#### 3.6.1 MoCA scale

**Figure 8:**
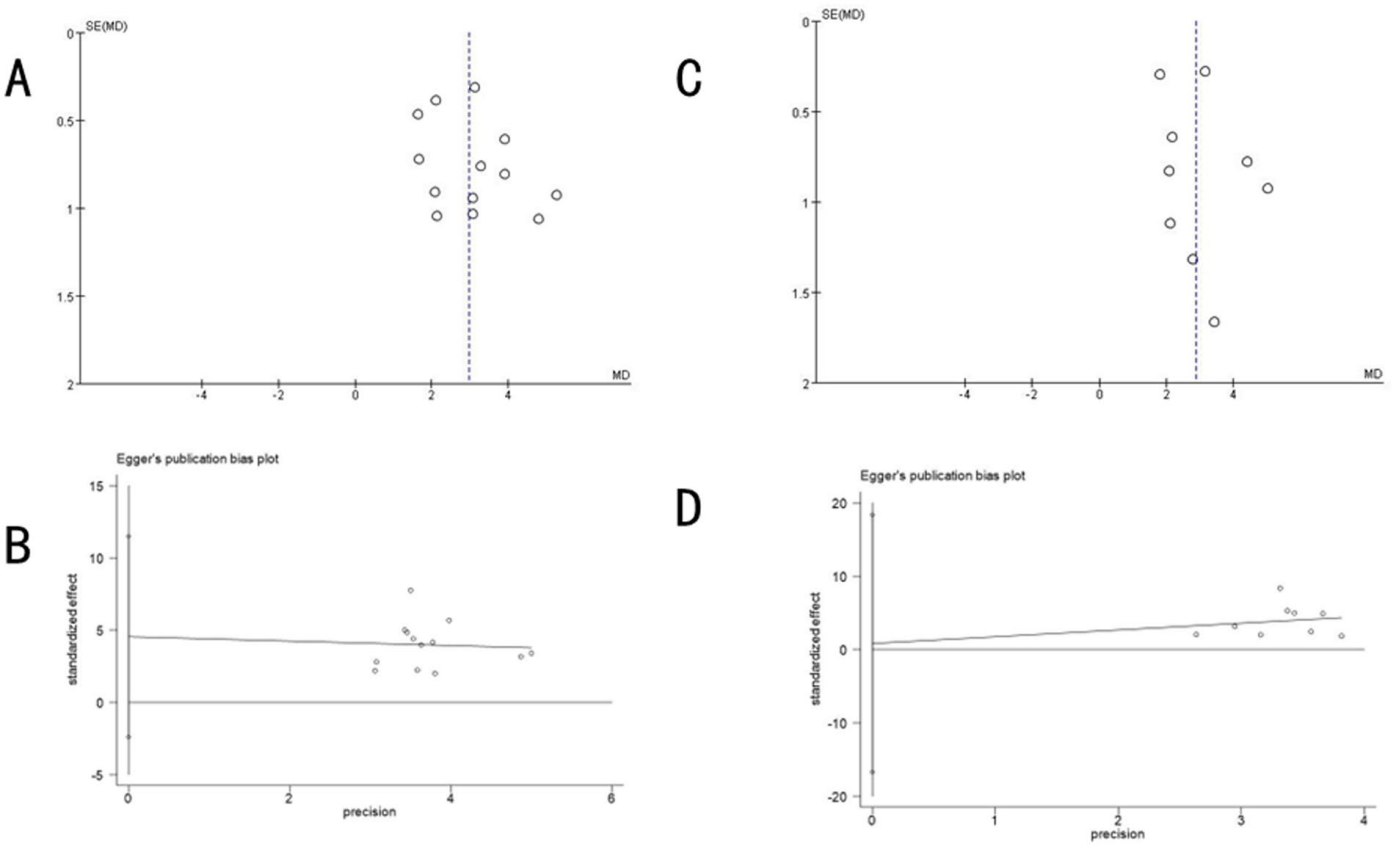
Funnel plot of publication bias **(A)** and Egger’s test plot **(B)** for the MoCA scale and publication bias **(C)** and Egger’s test plot **(D)** for the MMSE scale.

A funnel plot generated using RevMan 5.4 for MoCA scale outcome indicators displayed basic symmetry, suggesting a low likelihood of publication bias. Quantitative assessments via Begg’s test (*p* = 0.2997 > 0.05) and Egger’s test (*p* = 0.178 > 0.05) in Stata software further confirmed the absence of publication bias (Figure 8).

#### 3.6.2 MMSE scale

The funnel plot for MMSE scale outcomes showed mild asymmetry. However, Begg’s test (*p* = 0.4655 > 0.05) and Egger’s test (*p* = 0.914 > 0.05) indicated no significant publication bias (Figure 8).

### 3.7 Sensitivity analysis

**Figure 9:**
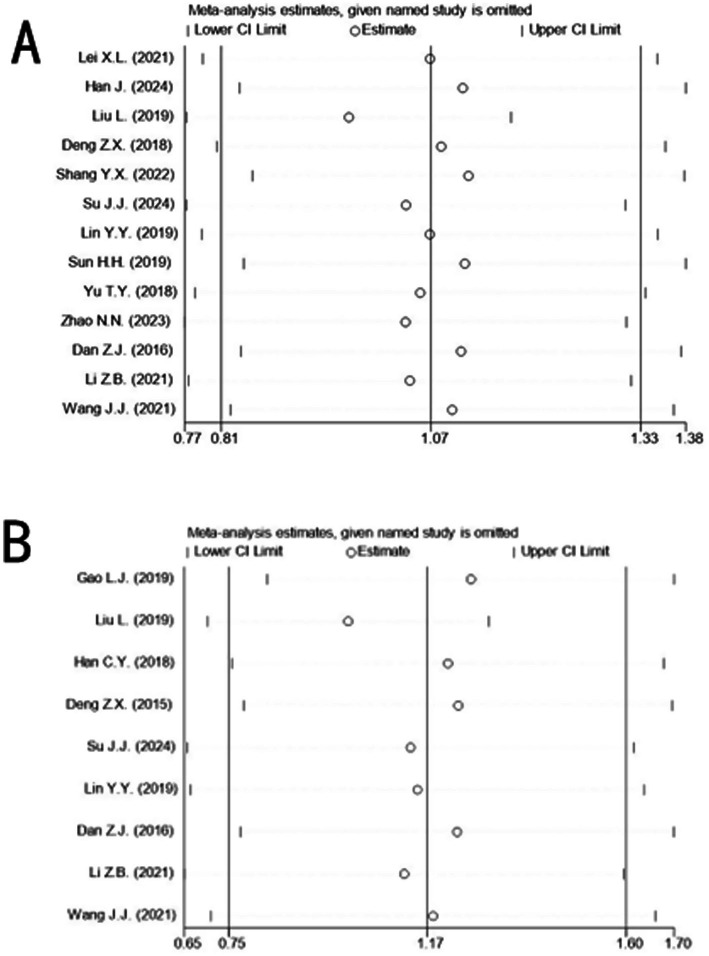
Sensitivity analysis of MoCA scale **(A)** and MMSE scale **(B)**.

Outcome indicators were sequentially excluded using RevMan 5.4, with no change in the overall direction of results, indicating the robustness of the meta-analysis findings. Further sensitivity analyses for MoCA and MMSE scales using Stata’s “metaninf module” demonstrated stable results, as excluding any single study did not alter the combined effect sizes beyond the 95% CI (Figure 9).

### 3.8 Trial sequential analysis (TSA)

#### 3.8.1 Clinical effectiveness rate

**Figure 10:**
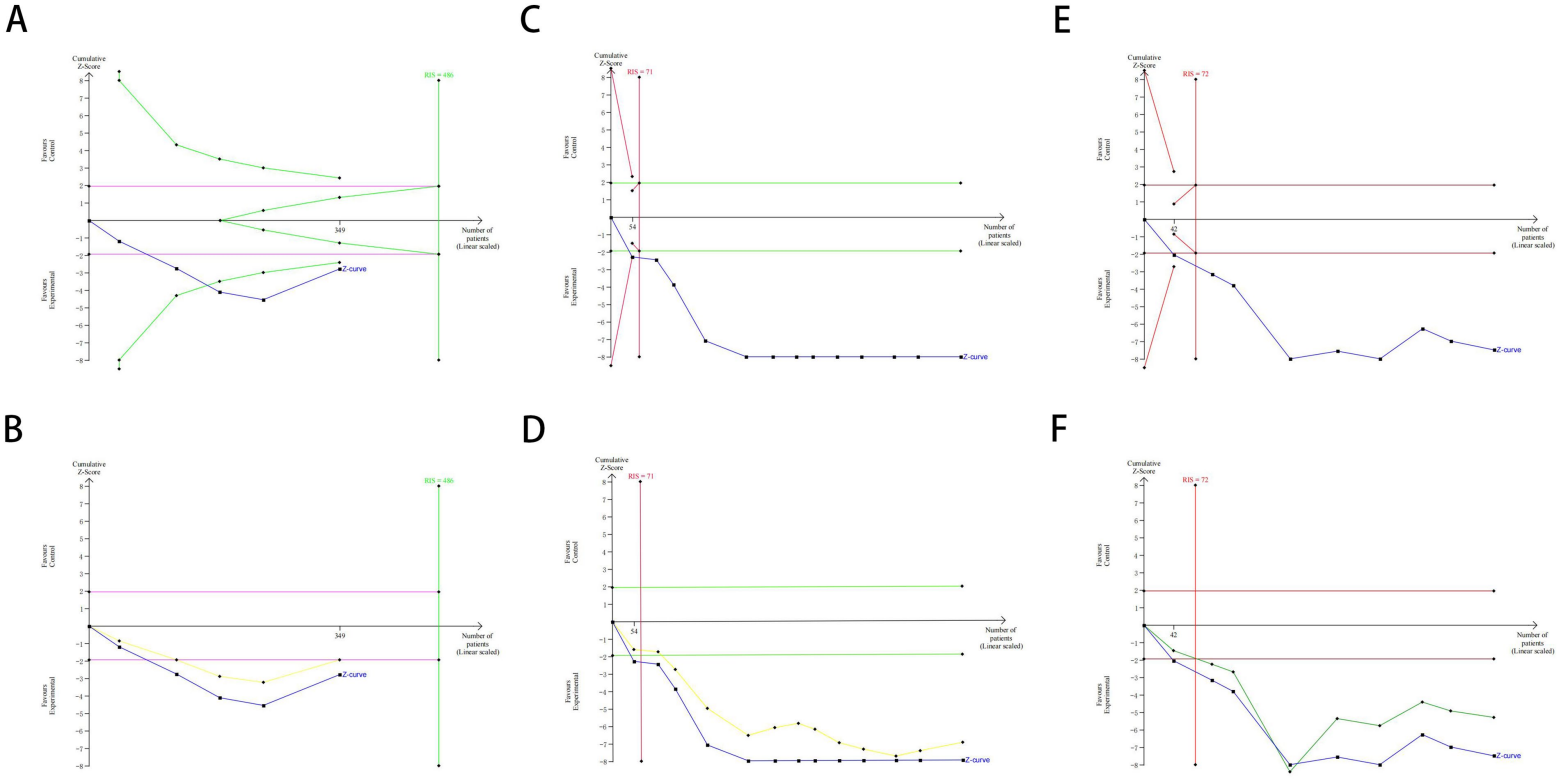
TSA chart of clinical effectiveness **(A)**, MoCA scale **(C)** and MMSE scale **(E)** and penalty statistic analysis chart of clinical effectiveness **(B)**, MoCA scale **(D)** and MMSE scale **(F)**.

Based on the relative risk reduction (RRR) = 1 − RR (28), combined with the meta-analysis results, the RRR was calculated to be −29%. The relative event rate in the control group = number of events in the control group / total number of events in the control group, yielding 74%. The efficacy meta-analysis included 5 trials with 349 cases, while the required information size (RIS) for the meta-analysis was 486. The results showed that the cumulative Z-value (solid blue line) crossed the traditional boundary (purple line; the horizontal line *Z* = 1.96 represents the conventional significance level, *α* = 0.05) after the inclusion of the second study and surpassed the trial sequential analysis (TSA) boundary (green line) after the inclusion of the third study. In the penalized statistics analysis, with a type I error set at 5% and a penalty value (*λ*) of 2, the penalized statistic (yellow line) exceeded the traditional boundary (purple line, *Z* = 1.96) after the inclusion of the second study. Although the cumulative sample size did not reach the RIS, further validation was deemed unnecessary, allowing an early definitive conclusion to be drawn (see Figure 10).

#### 3.8.2 MoCA scale

The meta-analysis of MoCA scores included 13 trials involving 869 patients, while the required information size (RIS) was calculated as 486. The results demonstrated that the cumulative Z-value (solid blue line) crossed both the traditional boundary (green line, *Z* = 1.96) and the trial sequential analysis (TSA) boundary (red line) after the inclusion of the first study, with the RIS achieved at just 71. Since the cumulative information size far exceeded the expected value, no further studies are needed to confirm that acupuncture combined with repetitive transcranial magnetic stimulation (rTMS) is superior to the control group in improving MoCA scores in patients with post-stroke cognitive impairment. To further validate the robustness of the findings, a penalized statistical analysis was performed with a type I error rate of 5% and a penalty factor (*λ*) of 2. The penalized statistic (yellow line) exceeded the traditional boundary (green line, *Z* = 1.96) after the inclusion of the third study, reinforcing the reliability of the conclusion. See Figure 10 for detailed results.

#### 3.8.3 MMSE scale

Based on the meta-analysis of MMSE scale scores, this study included 9 trials involving 492 cases, with a required information size (RIS) of 45 for the meta-analysis. The results demonstrated that the cumulative Z-value (blue solid line) crossed both the traditional threshold (purple solid line, *Z* = 1.96) and the trial sequential analysis (TSA) boundary (red solid line) after incorporating the first study. With the RIS achieved at 45 and the cumulative information size far exceeding the expected value, these findings conclusively indicate that no additional studies are needed to establish the superiority of acupuncture combined with repetitive transcranial magnetic stimulation in improving MMSE scores for post-stroke cognitive impairment patients compared to the control group. In the penalized analysis with a type I error set at 5% and a penalty value (*λ*) of 2, the adjusted statistical value (yellow solid line) exceeded the traditional threshold (purple solid line, *Z* = 1.96) after including the second study, further confirming the robustness of the conclusion (see Figure 10).

## 4 Discussion

### 4.1 Evidence summary and analysis

This study included sixteen randomized controlled trials (RCTs) involving 1,058 patients, employing multiple outcome measures to evaluate the clinical efficacy of acupuncture combined with repetitive transcranial magnetic stimulation (rTMS) in treating post-stroke cognitive impairment (PSCI). The assessed outcomes included clinical response rate, scores on the MMSE, MoCA, LOTCA, ADL, and MBI scales, serum levels of BDNF and NSE, as well as P300 potential latency and amplitude. The results demonstrated that various acupuncture modalities combined with rTMS were more effective than control interventions (such as conventional therapy alone including neuroprotective agents, antiplatelet therapy, and circulation-improving drugs or rTMS alone) in improving PSCI. Both qualitative and quantitative analyses supported this conclusion, and no significant publication bias was detected among the primary outcomes. However, only two studies reported adverse events: one observed no adverse effects, while the other documented mild dizziness. Due to the limited safety data, the long-term safety of this combined therapy remains uncertain, necessitating further high-quality RCTs to validate its safety profile in PSCI treatment.

The MMSE and MoCA scales are currently widely used clinical tools for cognitive function assessment. The MoCA scale addresses the limitations of the MMSE scale by incorporating evaluations of executive function, attention, and visuospatial abilities. However, the MoCA scale is influenced by educational level, and its cutoff values remain somewhat controversial (29). Combining these two scales as primary outcome measures helps compensate for their respective limitations. Subgroup analyses of the primary outcome measure (MoCA scale) based on treatment duration and intervention methods revealed that heterogeneity significantly decreased between studies with 2-week and 8-week treatment periods. Nevertheless, across different treatment course, the MoCA scores in the experimental groups were consistently higher than those in the control groups, with statistically significant differences. This suggests that acupuncture combined with repetitive transcranial magnetic stimulation (rTMS) improves cognitive function in PSCI patients regardless of treatment duration. Further subgroup analysis of different intervention methods showed that heterogeneity was substantially reduced in the electro-acupuncture, scalp acupuncture, and conventional acupuncture groups, indicating higher reliability of the results. In contrast, the Xingnao Kaiqiao acupuncture group exhibited significant heterogeneity, which may be attributed to various factors such as patient constitution, practitioner technique, and subjective assessment biases. Sensitivity analysis of the primary outcome measures (MoCA and MMSE scales) demonstrated that excluding specific studies did not significantly alter the high heterogeneity or the direction of the results, suggesting multiple complex sources of heterogeneity. Moreover, very low-certainty evidence suggests that Xingnao Kaiqiao acupuncture combined with rTMS may outperform the control group in improving MoCA scores. This uncertainty is likely due to study design limitations, including lack of allocation concealment, absence of blinding, and high heterogeneity. Developed by Academician Shi Xuemin in 1972, the Xingnao Kaiqiao acupuncture technique strictly defines acupoint location, needle insertion depth and direction, manipulation techniques, needling effects, and retention time (30). These stringent requirements may contribute to the lack of statistically significant clinical differences observed in some studies. The acupuncture interventions varied across studies, including electro-acupuncture, scalp acupuncture, and specialized techniques such as Xingnao Kaiqiao and Tongdu Xingshen. These variations may contribute to the observed heterogeneity, particularly in the Xingnao Kaiqiao subgroup, where technical precision and practitioner skill play critical roles.

Neuron-Specific Enolase (NSE) is specifically expressed in neuronal cells and plays a crucial role in glycolysis, significantly influencing neuronal metabolism (31). When neurons experience ischemia, hypoxia, or damage, the integrity of the cell membrane is disrupted, preventing NSE from binding to intracellular actin. This leads to neuronal injury or death, resulting in elevated NSE levels in cerebrospinal fluid (CSF) before its subsequent release into peripheral blood. Consequently, NSE serves as a biomarker for neuronal damage and neurodegenerative diseases, aiding in the assessment of brain injury. Clinically, it is widely used in the diagnosis of neurological and neuroendocrine disorders (32). Higher NSE levels correlate with more severe neurological impairment and poorer prognosis. In acute ischemic stroke patients, NSE levels can help evaluate thrombolytic therapy efficacy. Brain-Derived Neurotrophic Factor (BDNF) supports neuronal growth, synaptic plasticity, and inhibits apoptosis. Previous studies (33) have demonstrated that acupuncture enhances BDNF expression in animal models of focal cerebral ischemia, promoting dendritic spine regeneration and facilitating brain tissue repair. Recent animal studies provide direct mechanistic support for this synergy. For instance, Zhong (34) demonstrated in a rat model of cerebral ischemia that the combination of electroacupuncture and low-frequency rTMS significantly enhanced BDNF expression and promoted synaptic plasticity in the hippocampus, outperforming either intervention alone. Similarly, a 2024 study by Wu et al. found that combined therapy modulated the NCOA4-FTH1 pathway more effectively than monotherapies, reducing ferroptosis and protecting neuronal structure and function (6). These preclinical findings align with our clinical results, suggesting that the two modalities may act on complementary pathways—acupuncture potentially enhancing neurotrophic support and mitigating cellular stress, while rTMS promotes cortical excitability and network reorganization—ultimately leading to more robust cognitive recovery. Event-Related Potentials (ERPs) reflect the brain’s information-processing mechanisms and are closely associated with cognitive functions. These potentials indicate neural electrical activity in response to external stimuli, with P300 being a key ERP component. In Post-Stroke Cognitive Impairment (PSCI) patients, P300 latency is prolonged compared to healthy individuals, and its amplitude is inversely correlated with latency (35). This study suggests that acupuncture combined with repetitive transcranial magnetic stimulation (rTMS) improves cognitive function in PSCI patients, potentially by upregulating BDNF expression, reducing NSE levels, shortening P300 latency, and increasing amplitude. The Loewenstein Occupational Therapy Cognitive Assessment (LOTCA) evaluates cognitive function across seven domains, with higher scores indicating better cognitive performance. Meanwhile, the Modified Barthel Index (MBI) and Activities of Daily Living (ADL) scale assess functional independence. The MBI measures ten essential daily activities, with higher scores reflecting greater self-sufficiency (36). The ADL scale, scored out of 100, defines more than 60 as basic independence.

Neural functional recovery depends on neuroplasticity, where external training or stimulation enables post-stroke neurons and their networks to adapt to new changes, promoting functional restoration. Repetitive transcranial magnetic stimulation (rTMS) induces action potentials, activates neural circuits, and enhances cerebral metabolism while modifying neurotransmitter systems and synaptic connections. These neurobiological changes optimize goal-directed behavior control networks, ultimately improving cognitive function (37).

Acupuncture has been demonstrated to ameliorate post-stroke cognitive impairment (PSCI) by modulating immune responses through signal pathways and cytokines, thereby influencing neuronal function (38, 39). According to the interhemispheric inhibition model, stroke disrupts the dynamic balance between cerebral hemispheres, resulting in dual inhibition that complicates recovery. Repetitive transcranial magnetic stimulation (rTMS) can promote neural reorganization in the central nervous system by regulating cellular signaling pathways. This intervention enhances neural plasticity and reestablishes balanced excitability, ultimately improving cognitive function (40).

### 4.2 Strengths and limitations

In future clinical practice, there is growing emphasis on comprehensive therapies for the intervention and treatment of patients with post-stroke cognitive impairment (PSCI). The combination of acupuncture and repetitive transcranial magnetic stimulation (rTMS) has emerged as an effective and safe clinical approach. However, rTMS has not yet been widely adopted and is currently only available in tertiary hospitals in China. As a result, there are limited reports on the combined use of acupuncture and rTMS. This study aims to provide a systematic evaluation, offering a safe and effective method for the clinical treatment of PSCI.

Compared to traditional meta-analyses, this study innovatively incorporates trial sequential analysis (TSA) to further assess whether the current sample size is sufficient to draw robust conclusions, thereby providing a reference for the design of future clinical trials. Additionally, subgroup analysis and sensitivity analysis were employed to explore the sources of heterogeneity in primary outcome measures. Both qualitative and quantitative methods were used to evaluate publication bias, enhancing the credibility of the findings.

However, certain limitations should be acknowledged. For instance, inadequate descriptions of randomization in some studies may introduce selection bias. Only one study mentioned blinding, while the lack of transparency in others raises concerns, particularly since the effects of acupuncture may be influenced by the expectations of both patients and practitioners. High heterogeneity among the included studies may stem from variations in acupuncture point selection, differences in conventional rehabilitation therapies used in control groups, and inconsistencies in rTMS parameters (including frequency, coil placement, and stimulation intensity). The insufficient reporting of these technical details precluded planned sensitivity or subgroup analyses based on rTMS protocols, which is a significant limitation of the current evidence base. Furthermore, all included studies were published in Chinese journals, with no international publications, which may contribute to publication bias. The geographical distribution of the included trials is a notable limitation. All studies were conducted in China and published in Chinese journals, which may limit the generalizability of our findings to other ethnic populations and healthcare systems outside of China. This concentration introduces potential for geographical and language bias, as effective interventions may be influenced by genetic background, cultural factors, and variations in standard care. Furthermore, while our sensitivity analysis excluding studies with poorly described randomization methods did not alter the primary conclusions, the overall methodological quality of the included studies, particularly regarding allocation concealment and blinding, was often suboptimal. These factors urge caution in extrapolating the results and highlight the necessity for future multi-center, international RCTs conducted with rigorous methodology to confirm the efficacy and generalizability of acupuncture combined with rTMS for PSCI. The GRADE assessment indicated that some studies were of low quality, potentially affecting the results. While reported AE rates were low (1.2%), systematic reviews of acupuncture report needle-site reactions in 3–8% of cases. We recommend future trials use CTCAE criteria, particularly for: Acupuncture: needle pain, hematomar TMS: headache, scalp discomfort.

Even if the combined therapy shows compelling clinical benefits, its cost-effectiveness relative to monotherapies remains unclear. Future studies must integrate economic metrics to guide evidence-based decisions, especially in resource-constrained settings. Until then, the combined approach may be most justifiable for patients with moderate-to-severe PSCI unresponsive to monotherapies. Despite observing substantial heterogeneity in some outcomes, we did not perform meta-regression to explore potential sources due to the limited number of studies per outcome. Future updates with more trials may allow for such analyses. Future research should focus on well-designed, rigorous, and feasible studies with standardized protocols and larger sample sizes to further validate these findings. Such efforts would provide a stronger theoretical foundation for the use of acupuncture combined with rTMS in treating PSCI.

### 4.3 Future outlook

While the current evidence base relies heavily on subjective cognitive assessments such as MoCA and MMSE, recent advances in neuroimaging and electrophysiological techniques provide promising avenues for objectively evaluating the therapeutic effects of this combined approach. Electroencephalography (EEG) and functional magnetic resonance imaging (fMRI) have emerged as powerful tools to elucidate the neural mechanisms underlying acupuncture and rTMS interventions. Recent research has confirmed that these neuroimaging techniques can significantly assist in evaluating the etiology and prognosis of cognitive impairment (41). For instance, quantitative EEG analysis has demonstrated that acupuncture modulates oscillatory activity across multiple frequency bands, particularly enhancing alpha and theta power associated with cognitive processing (42). Furthermore, advanced decoding methods such as neural manifold representation learning have successfully identified distinct acupuncture-induced brain states, revealing specific neural representations in sensorimotor and frontal regions (43).

The integration of acupuncture and rTMS appears to produce synergistic effects through complementary neural pathways. Neuroimaging evidence shows that acupuncture enhances neurovascular coupling and promotes hemodynamic responses in key regions including the insula, anterior cingulate cortex, and prefrontal cortex (6), while rTMS strengthens functional connectivity within critical networks such as the frontoparietal network (43). This combined approach may promote cognitive recovery through multiple mechanisms: enhancing neuroplasticity via BDNF-mediated dendritic remodeling, reestablishing interhemispheric balance disrupted by stroke, and optimizing neural circuit dynamics through complementary modulation of cortical activity. Notably, EEG studies have demonstrated that acupuncture can modulate aperiodic neural activity characteristics reflecting excitatory/inhibitory balance (44), an effect that complements rTMS’s influence on cortical oscillatory rhythms.

Future research should prioritize exploring the spatiotemporal dynamics of brain activity using multimodal approaches. The work by Yu et al. (45, 46) exemplifies this direction, employing EEG detection systems to assess regulatory effects through spectral power and aperiodic exponent adjustments, and investigating brain responses under different acupuncture manipulations. These quantitative methods offer significant potential for developing objective biomarkers of treatment efficacy and facilitating personalized therapeutic strategies. As acupuncture has been shown to modulate spatiotemporal brain activity and improve cognitive function in neurological disorders (47), future studies focusing on EEG and neuroimaging mechanisms of the combined therapy will be crucial for advancing evidence-based, personalized approaches to PSCI management.

## 5 Conclusion

This study demonstrates that the combination of acupuncture and repetitive transcranial magnetic stimulation (rTMS) can enhance cognitive function in patients with post-stroke cognitive impairment (PSCI), potentially through mechanisms involving the remodeling of damaged neuronal structures in the brain. However, the evidence supporting specific acupuncture (such as Xingnao Kaiqiao) combined with rTMS for improving Montreal Cognitive Assessment (MoCA) scores remains insufficient and requires further validation. While the combined therapy shows compelling clinical benefits, its cost-effectiveness relative to monotherapies remains unclear. Future studies must integrate economic metrics to guide evidence-based decisions, especially in resource-constrained settings. Until then, the combined approach may be most justifiable for patients with moderate-to-severe PSCI unresponsive to monotherapies. These findings highlight the need for standardized clinical protocols in applying this specific acupuncture method, as well as higher-quality, rigorously designed randomized controlled trials (RCTs) to provide more robust evidence for its efficacy.

## Data Availability

The original contributions presented in the study are included in the article/Supplementary material, further inquiries can be directed to the corresponding author.
