# Supplementary material for: Acupuncture combined with repetitive transcranial magnetic stimulation for the treatment of post-stroke cognitive impairment: a systematic review and meta-analysis with trial sequential analysis
Source: Front Neurol. 2025 Dec 12;16:1663452. doi: 10.3389/fneur.2025.1663452 (PMC12742397; doi:10.3389/fneur.2025.1663452)
Supplement: Supplementary file 1 [file Data_Sheet_1.docx]

**1 Pubmed 14**

**#1** "Stroke"[Mesh]OR “Strokes”[Title/Abstract]OR “Cerebrovascular Accident”[Title/Abstract]OR“Cerebrovascular Accidents”[Title/Abstract]OR”Cerebral Stroke”[Title/Abstract]OR”Cerebral Strokes”[Title/Abstract] OR “Cerebrovascular Apoplexy”[Title/Abstract]OR”Brain Vascular Accident”[Title/Abstract] OR “Brain Vascular Accidents”[Title/Abstract])OR “Cerebrovascular Stroke”[Title/Abstract]OR”Cerebrovascular Strokes”[Title/Abstract] OR “Apoplexy”[Title/Abstract]OR “CVA Cerebrovascular Accident”[Title/Abstract]OR “CVAs Cerebrovascular Accident”[Title/Abstract]OR”Acute Stroke”[Title/Abstract]OR “Acute Strokes”[Title/Abstract] OR “Acute Cerebrovascular Accident”[Title/Abstract] OR “Acute Cerebrovascular Accidents”[Title/Abstract]

**#2** "Cognitive Dysfunction"[Mesh]) OR (Cognitive Dysfunctions[Title/Abstract])) OR (Cognitive Disorder[Title/Abstract])) OR (Cognitive Disorders[Title/Abstract])) OR (Cognitive Impairments[Title/Abstract])) OR (Cognitive Impairment[Title/Abstract])) OR (Mild Cognitive Impairment[Title/Abstract])) OR (Mild Cognitive Impairments[Title/Abstract])) OR (Cognitive Decline[Title/Abstract])) OR (Cognitive Declines[Title/Abstract])) OR (Mental Deterioration[Title/Abstract])) OR (Mental Deteriorations[Title/Abstract])))

**#3** (((("Acupuncture"[Mesh]) OR (Pharmacopuncture[Title/Abstract])) OR (Transcranial Magnetic Stimulation[Title/Abstract])) OR (Transcranial Magnetic Stimulations[Title/Abstract])))

**#4** (("Randomized Controlled Trials as Topic"[Mesh]) OR (Randomized Controlled Trial[Publication Type]))

**2 CNKI 356**

（主题：卒中 + 脑卒中 + 出血性卒中 + 脑梗死(精确) + 脑出血） AND （（主题：认知障碍 + 认知功能障碍） AND （（主题：针刺 + 针灸 + 电针 + 耳针 + 体针 + 头针 + 头皮针(精确)）+ 穴位埋线 + 艾灸 + 经颅磁刺激） AND （（摘要：随机对照试验）OR（摘要：随机对照实验）OR（摘要：随机对照研究）OR（摘要：RCT）OR（摘要：随机对照）OR（摘要：随机））

**3 Wanfang Data 53**

( 主题:(脑卒中 or 认知障碍 or 卒中后认知障碍) or 题名或关键词:(脑卒中 or 认知障碍 or 卒中后认知障碍) or 摘要:(脑卒中 or 认知障碍 or 卒中后认知障碍)) and( 主题:(针灸 and 经颅磁刺激) or 题名或关键词:(针灸 and 经颅磁刺激) or 摘要:(针灸 and 经颅磁刺激))

**4 VIP 32**

M=(卒中后认知障碍 OR 脑卒中 OR 认知障碍 OR 认知功能障碍 OR 中风) AND M=（针灸 AND 经颅磁刺激）

**5 WOS 256**

1.TS=(Stroke OR Strokes OR Cerebrovascular Accident OR Cerebrovascular Accidents OR Cerebral Stroke OR Cerebral Strokes OR Cerebrovascular Apoplexy OR Brain Vascular Accident OR Brain Vascular Accidents OR Cerebrovascular Stroke OR Cerebrovascular Strokes OR Apoplexy OR CVA Cerebrovascular Accident OR CVAs Cerebrovascular Accident OR Acute Stroke OR Acute Strokes OR Acute Cerebrovascular Accident OR Acute Cerebrovascular Accidents)

AND TS=(Cognitive Dysfunction OR Cognitive Dysfunctions OR Cognitive Disorder OR Cognitive Disorders OR Cognitive Impairments OR Cognitive Impairment OR Mild Cognitive Impairment OR Mild Cognitive Impairments OR Cognitive Decline OR Cognitive Declines OR Mental Deterioration OR Mental Deteriorations)

AND TS=(Acupuncture OR Pharmacopuncture OR Transcranial Magnetic Stimulation OR Transcranial Magnetic Stimulations)

AND TS=(Randomized Controlled Trials OR Controlled Clinical Trial)

2.TS=(Stroke OR Strokes OR Cerebrovascular Accident OR Cerebrovascular Accidents OR Cerebral Stroke OR Cerebral Strokes OR Cerebrovascular Apoplexy OR Brain Vascular Accident OR Brain Vascular Accidents OR Cerebrovascular Stroke OR Cerebrovascular Strokes OR Apoplexy OR CVA Cerebrovascular Accident OR CVAs Cerebrovascular Accident OR Acute Stroke OR Acute Strokes OR Acute Cerebrovascular Accident OR Acute Cerebrovascular Accidents) AND TS=(Cognitive Dysfunction OR Cognitive Dysfunctions OR Cognitive Disorder OR Cognitive Disorders OR Cognitive Impairments OR Cognitive Impairment OR Mild Cognitive Impairment OR Mild Cognitive Impairments OR Cognitive Decline OR Cognitive Declines OR Mental Deterioration OR Mental Deteriorations) AND TS=(Acupuncture OR Pharmacopuncture OR Transcranial Magnetic Stimulation OR Transcranial Magnetic Stimulations) AND TS=(Randomized Controlled Trials OR Controlled Clinical Trial)

**6 Cochrane Library 84**

#1 MeSH descriptor:Stroke

#2 Ti Ab Keyword Strokes OR Cerebrovascular Accident OR Cerebrovascular Accidents OR Cerebral Stroke OR Cerebral Strokes OR Cerebrovascular Apoplexy OR Brain Vascular Accident OR Brain Vascular Accidents OR Cerebrovascular Stroke OR Cerebrovascular Strokes OR Apoplexy OR CVA Cerebrovascular Accident OR CVAs Cerebrovascular Accident OR Acute Stroke OR Acute Strokes OR Acute Cerebrovascular Accident OR Acute Cerebrovascular Accidents

#3 #1 or #2

#4 MESH Cognitive Dysfunction

#5 **Ti Ab Keyword Strokes** Cognitive Dysfunctions OR Cognitive Disorder OR Cognitive Disorders OR Cognitive Impairments OR Cognitive Impairment OR Mild Cognitive Impairment OR Mild Cognitive Impairments OR Cognitive Decline OR Cognitive Declines OR Mental Deterioration OR Mental Deteriorations

#6 #4 or #5

#7 #3 and #6

#8 MESH Acupuncture

#9 **Ti Ab Keyword Strokes** Pharmacopuncture OR Transcranial Magnetic Stimulation OR Transcranial Magnetic Stimulations

#10 #8 or #9

#11 #7 and #10

#12 **Ti Ab Keyword** Strokes Randomized Controlled Trials OR Controlled Clinical Trial

#13 #11 and #12

**7 CBM 47**

("卒中后认知障碍"[摘要:智能] OR "脑卒中"[摘要:智能] OR "认知障碍"[摘要:智能])AND("针灸"[摘要:智能] AND "经颅磁刺激"[摘要:智能])

**8 Embase 55**

#1 'stroke'/exp OR 'stroke' OR 'strokes' OR 'cerebrovascular accident'/exp OR 'cerebrovascular accident' OR 'cerebrovascular accidents' OR 'cerebral stroke'/exp OR 'cerebral stroke' OR 'cerebral strokes' OR 'cerebrovascular apoplexy' OR 'brain vascular accident'/exp OR 'brain vascular accident' OR 'brain vascular accidents' OR 'cerebrovascular stroke' OR 'cerebrovascular strokes' OR 'apoplexy'/exp OR 'apoplexy' OR 'cva cerebrovascular accident' OR 'cvas cerebrovascular accident' OR 'acute stroke'/exp OR 'acute stroke' OR 'acute strokes' OR 'acute cerebrovascular accident' OR 'acute cerebrovascular accidents'

#2 'cognitive dysfunction' OR 'cognitive dysfunctions' OR 'cognitive disorder' OR 'cognitive disorders' OR 'cognitive impairments' OR 'cognitive impairment' OR 'mild cognitive impairment' OR 'mild cognitive impairments' OR 'cognitive decline' OR 'cognitive declines' OR 'mental deterioration' OR 'mental deteriorations'

#3 'acupuncture' OR 'pharmacopuncture' OR 'transcranial magnetic stimulation' OR 'transcranial magnetic stimulations'

#4 'randomized controlled trials' OR 'controlled clinical trial'

#5 #1 and #2 and #3 and #4
